# Functional polymorphisms in matrix metalloproteinases -1, -3, -9 and -12 in relation to cervical artery dissection

**DOI:** 10.1186/1471-2377-9-40

**Published:** 2009-08-09

**Authors:** Armin Buss, Katrin Pech, Susanne Roelver, Brunhilde Bloemeke, Christoph Klotzsch, Sebastian Breuer

**Affiliations:** 1 Department of Neurology, Aachen University Medical School, Pauwelsstrasse 30, Aachen, Germany; 2 Department of Ecotoxicology and Toxicology, University of Trier, Am Wissenschaftspark 27-29, Trier, Germany; 3 Department of Neurology, Schmieder-Kliniken, Zum Tafelholz 8, Allensbach, Germany

## Abstract

**Background:**

Cervical artery dissection is a leading cause of cerebral ischemia in young adults. Morphological investigations have shown alterations in the extracellular matrix (ECM) of affected vessel walls. As matrix metalloproteinases (MMP) play a central role in the regulation of the ECM, an increased expression of these enzymes might lead to the endothelial damage in spontaneous cervical artery dissection (sCAD). Five different DNA polymorphisms in MMP-1, -3, -9 and -12 were tested for their frequency in patients with sCAD and compared with those of a control population.

**Methods:**

Blood was sampled from 70 unrelated patients presenting consecutively in the department of neurology of the Aachen University Medical School with sCAD and from 87 control subjects living in the same area as the patients. The MMP polymorphisms were analyzed with hybridization probes using the LightCycler™ (Roche Diagnostics), by sequencing using the ABI 310 Genetic Analyzer (Applied Biosystems) and with the GeneScan program on a ABI 310 Genetic Analyzer.

**Results:**

No statistically significant differences in the allelic distribution were found between sCAD patients and the controls.

**Conclusion:**

Alleles of these 5 functional polymorphisms of MMPs seem not to be associated with structural alterations in the blood vessel wall of sCAD patients. However, this does not exclude a pathogenetic role for MMPs in sCAD via secondary factors such as cytokines that are able to induce these enzymes in cervical blood vessel walls.

## Background

Spontaneous cervical artery dissection (sCAD) is a leading cause of cerebral ischemia in young adults [1]. The incidence in the general population is estimated at 2.6 to 2.9 per 100 000 and the mean age of occurrence is 44 to 46 years [2,3]. The reason why these sCADs occur in otherwise healthy-appearing young adults remains poorly understood although numerous risk factors have been postulated such as recent infection [4], hyperhomocysteinemia [5,6], low levels of α1-antitrypsin [7] and hypertension [8]. For most factors, evidence is limited either due to small sample size or conflicting data from different studies [9]. Several arguments suggest a genetic influence in sCAD [10]. Two studies reported the overall presence of a family history of sCAD in 2% to 3% of patients. Secondly, patients with sCAD often present with concomitant arterial abnormalities such as fibromuscular dysplasia or aortic root dilation. Finally, morphological investigations have consistently shown alterations in the extracellular matrix (ECM) of skin biopsies even in patients without known connective tissue disorders. Hereditary monogenic disorders such as vascular Ehlers-Danlos syndrome are rare in sCAD [11,12]. Genetic factors might however play a role as part of a multifactorial predisposition (eg, causing a constitutional weakness of the vessel wall or predisposing the vessel wall to inflammatory processes) [10]. Matrix metalloproteinases (MMP) are interesting candidate genes as they play a central role in the regulation of the ECM and earlier studies in vascular pathologies have already shown evidence for an increased activity of MMPs in the wall of affected blood vessels in aortic dissection [13]. In sCAD patients, a recent study reported higher plasma levels of MMP-2 compared to patients with an ischemic stroke from different reasons [14]. In the present study, we therefore investigated 5 polymorphisms in the promoter region of the MMP genes with a known increased expression rate of the corresponding enzyme as possible risk factors for sCAD [15-19].

## Methods

In this single centre study, blood was sampled from 70 consecutive unrelated patients who presented with sCAD in the years 1999 to 2003 in the department of neurology of the Aachen University Medical School. The patients comprised 42 men and 28 women (age range 28–70 years; median 48.5 years). The diagnosis of sCAD was based on magnetic resonance imaging angiography and/or digital substraction angiography. Fifty-four patients suffered a dissection of the carotid artery, 16 of the vertebral artery; no cases with multiple cervical artery dissections were included. Patients with known connective tissue disorders such as Ehlers-Danlos syndrome, angiographic signs of fibromuscular dysplasia or assumed traumatic cervical artery dissection were excluded. Eighty-seven german caucasian control subjects of similar age and sex distribution living in the same area as the patients were randomly selected in the Aachen University Blood Bank. The control group comprised 50 men and 37 women (age range 22–68 years; median 48 years). The study was approved by the Aachen University Ethical Committee and written consent was obtained from all participants.

DNA was isolated from EDTA blood using the QIAamp DNA Blood kit (Qiagen, Hilden, Germany). The MMP-1 (-1607 1G/2G) polymorphism (rs1799750) was analyzed with hybridization probes using the LightCycler™ (Roche Diagnostics). Primer and probes were designed by Tib Molbiol (MMP1-Sensor: 5'-GAT TTG AGA ATA AGT CAT AT CTT TCT AAT X, MMP1-Anker: 5'-LC Red640- TTA ACT ACA ATT TCC TCA TCT AAG TGG C p, MMP1proS: 5'-TTC TTA CCC TCT TGA ACT CAC ATG-3', MMP1proAS: 5'-TTC CTC CCC TTA TGG ATT CC-3'). The MMP-3 (-1612 5A/6A, rs3025058), -9 (-1562 C/T, rs3918242) and -12 (-82 A/G, rs2276109) polymorphisms were analyzed by sequencing using the ABI 310 Genetic Analyzer (Applied Biosystems) with a standard protocol. The multiallelic (CA)n microsatellite polymorphism in the MMP-9 gene was analyzed with the GeneScan program on a ABI 310 Genetic Analyzer (FAM-MMP9F: 5'-GAC TTG GCA GTG GAG ACT GCG GGC A-3', MMP9R: 5'-GAC CCC ACC CCT CCT TGA CAG GCA-3'). To avoid inconsistencies between different runs, the 6 most common CA-repeats were cloned, sequenced and used as an internal standard in every run. For statistical analyses, χ^2 ^test was used to determine if the genotypes were in the Hardy-Weinberg equilibrium. Odds ratio and the 95% confidence interval were calculated to test the association of genotype and sCAD.

## Results

The results of the genetic analyses are presented in Table 1 and 2. There were no statistically significant differences between patients and controls in the 5 polymorphisms investigated. The genotype distributions for all polymorphisms were compatible with those expected from the Hardy-Weinberg equilibrium, except for MMP-12 in patients.

**Table 1:** Distribution of Genotypes and Alleles of all single nucleotide polymorphisms

			Controls, No (Frequency)	Patients, No (Frequency)	Odds ratio (95% confidence interval)
MMP-1	Genotype	2G/2G	22 (0.253)	15 (0.214)	Reference
		2G/1G	50 (0.575)	34 (0.486)	1.003 (0.456 to 2.204)
		1G/1G	15 (0.172)	21 (0.3)	0.487 (0.191 to 1.237)
		2G/2G	22 (0.253)	15 (0.214)	Reference
		1G/1G or 1G/2G	66 (0,747)	55 (0,786)	0,818 (0,39 to 1,73)
		1G/1G	15 (0.172)	21 (0.3)	Reference
		2G/2G or 2G/1G	72 (0.828)	49 (0.7)	2,05 (0.97 to 4.38)
		2G/2G or 2G/1G	72 (0.828)	49 (0.7)	Reference
		1G/1G	15 (0.172)	21 (0.3)	0.486 (0.23 to 1.03)
		1G/1G or 1G/2G	66 (0,747)	55 (0,784)	Reference
		2G/2G	22 (0,253)	15 (0,214)	1,222 (0.579 to 2.581)
		1G/2G	50 (0.575)	34 (0.486)	Reference
		2G/2G or 1G/1G	37 (0,425)	36 (0,514)	0,6989 (0.371 to 1.316)
MMP-3	Genotype	6A/6A	21 (0.242)	16 (0.228)	Reference
		5A/6A	47 (0.540)	34 (0.486)	0.359 (0.149 to 0.865)
		5A/5A	19 (0.218)	20 (0.286)	0.724 (0.293 to 1.787)
		6A/6A	21 (0.242)	16 (0.228)	Reference
		5A/5A or 6A/5A	66 (0,758)	54 (0,772)	0,931 (0.443 to 1.958)
		5A/5A	19 (0.218)	20 (0.286)	Reference
		6A/6A or 6A/5A	68 (0.782)	50 (0.714)	1,432 (0.692 to 2.959)
		6A/5A	47 (0.540)	34 (0.486)	Reference
		6A/6A or 5A/5A	40 (0,460)	36 (0,514)	0,8038 (0.428 to 1.510)
		5A/5A or 6A/5A	66 (0,758)	54 (0,772)	Reference
		6A/6A	21 (0.242)	16 (0.228)	1,074 (0.511 to 2.258
		6A/6A or 6A/5A	68 (0.782)	50 (0.714)	Reference
		5A/5A	19 (0.218)	20 (0.286)	0.698 (0.338 to 1.444)
MMP-9	Genotype	T/T	1 (0.011)	0 (-)	
		C/T	27 (0.310)	20 (0.286)	
		C/C	59 (0.678)	50 (0.714)	
		T/T or T/C	28 (0.322)	20 (0.286)	Reference
		C/C	59 (0.678)	50 (0.714)	0.843 (0.424 to 1.675)
		TC	27 (0.310)	20 (0.286)	Reference
		C/C or T/T	60 (0,690)	50 (0.714)	0,8889 (0.446 to 1.771)
		C/C	59 (0.678)	50 (0.714)	Reference
		T/C or T/T	28 (0.322)	20 (0.286)	1,1864 0.5972 to 2.357
MMP-12	Genotype	A/A	72 (0.828)	48 (0.686)	Reference
		A/G	14 (0.161)	22 (0.314)	0.424 (0.198 to 0.909)
		G/G	1 (0.011)	0 (-)	
		A/G	14 (0.161)	22 (0.314)	Reference
		A/A or G/G	73 (0,839)	48 (0.686)	2,389 (1.115 to 5.13)
		A/A	72 (0.828)	48 (0.686)	Reference
		G/G or A/G	15 (0,172)	22 (0.314)	0,4545 (0.215 to 0.963)
		G/G or A/G	15 (0,172)	22 (0.314)	Reference
		A/A	72 (0.828)	48 (0.686)	2,2 (1.038 to 4.662)
		A/A or G/G	73 (0,839)	48 (0.686)	Reference
		A/G	14 (0.161)	22 (0.314)	0,4184 (0.195 to 0.897)

**Table 2:** Allele frequencies of the MMP-9 multiallelic (CA)n microsatellite polymorphism

Allele	No. of CA Repeats	Controls, No (Frequency)	Patients, No (Frequency)	Odds ratio (95% confidence interval)
1	12	0 (-)	1 (0.007)	

2	13	2 (0.011)	5 (0.036)	

3	14	96 (0.551)	67 (0.478)	0.746 (0.477 to 1.117)

4	15	6 (0.034)	3 (0.021)	

5	16	0 (-)	1 (0.007)	

6	19)	2 (0.011)	4 (0.028)	

7	20	1 (0.006)	5 (0.036)	

8	21	19 (0.109)	23 (0.164)	1.604 (0.834 to 3.082)

9	22	26 (0.149)	20 (0.142)	0.949 (0.505 to 1.782)

10	23	19 (0.109)	10 (0.071)	0.628 (0.281 to 1.397)

11	24	3 (0.017)	1 (0.007)	

Total		174	140	

## Discussion

MMPs are a family of extracellular zinc- and calcium-dependent endopeptidases that degrade the extracellular matrix and other extracellular proteins [20]. The 23 mammalian MMPs can be placed into sub-groups based on structural similarities and substrate specificity and they are capable of degrading virtually all extracellular proteins. In the present study, 5 polymorphisms in the promoter region of MMP-1, -3, -9 and -12 genes were investigated for a possible genetic association with sCAD. All polymorphisms lead to an increased expression of the corresponding enzyme and might therefore lead to ECM instability in blood vessel walls [15-19]. In an earlier study investigating polymorphisms in the MMP-9 gene, no differences in the allelic distribution of both polymorphisms were found between sCAD patients and controls [21]. In accordance with these results, we could also not detect statistically significant differences in overall frequencies of both the single nucleotide polymorphism and the dinucleotide repeat length polymorphism of the MMP-9 gene. Furthermore, concerning the latter polymorphism, its most active variant of 23 dinucleotide repeats was more frequently found in the control group. The frequencies of the single nucleotide polymorphisms of the MMP-1, -3 and -12 genes which were not previously investigated in sCAD patients were also comparable between the patients and the control group.

The present study did not find a statistically significant association between MMP polymorphisms and sCAD. Due to the relatively small sample size of 70 patients, these negative findings might however be due to insufficient power. As the present results are not sufficient to make a definite statement about the genetic association they need to be replicated in a larger group of patients. This is however hardly possible in single centre studies due to the overall low prevalence of sCAD and will need future multicenter investigations.

## Conclusion

The results of the present study do not support the hypothesis that polymorphisms in MMP-1, -3, -9 and -12 leading to an increased expression rate of the corresponding enzyme are susceptibility factors of sCAD. However, this does not exclude a pathogenetic role for MMPs in sCAD via secondary factors such as cytokines that are able to induce these enzymes in cervical blood vessel walls. Secondly, future multicenter studies including larger sample sizes are needed to verify or dismiss the lack of genetic association of the MMP polymorphisms and sCAD found in this pilot trial.

## Abbreviations

sCAD: spontaneous cervical artery dissection; ECM: Extracellular Matrix; MMP: Matrix Metalloproteinase.

## Competing interests

The authors declare that they have no competing interests.

## Authors' contributions

AB designed and coordinated the study and drafted the manuscript. KP and SK carried out the analysis of the polymorphisms. BB participated in the design of the study and helped to draft the manuscript. CK participated in the design of the study, provided specimens and helped to draft the manuscript. SB designed and coordinated the study and drafted the manuscript. All authors read and approved the final manuscript.

## Pre-publication history

The pre-publication history for this paper can be accessed here:


